# Tumor cell dormancy as an adaptive cell stress response mechanism

**DOI:** 10.12688/f1000research.12174.1

**Published:** 2017-12-14

**Authors:** Laura Vera-Ramirez, Kent W. Hunter

**Affiliations:** 1 Laboratory of Cancer Biology and Genetics, Center for Cancer Research, National Cancer Institute, Bethesda, MD, USA

**Keywords:** tumor cell dormancy/MAPK/Src kinase/unfolded protein response/autophagy/hypoxia

## Abstract

Metastases are responsible for most cancer-related deaths. The kinetics of tumor relapse is highly heterogeneous, ranging from recurrences shortly after diagnosis to years or even decades after the initial treatment. This subclinical period is known as tumor dormancy, in which residual disease remains in an undetectable state before finally appearing as an overtly proliferative metastasis. Despite recent advances in our understanding of the molecular mechanisms leading to tumor dormancy, it is still a poorly understood phase of cancer progression, which limits opportunities for the design of successful therapeutic interventions. The influence of the tumor microenvironment at the metastatic site and anti-metastatic immune responses have been shown to play a crucial role in the onset and maintenance of metastatic dormancy. However, there is still a significant gap in our understanding of how dormant cells remain viable in a quiescent state for long periods of time. Here, we review the latest experimental evidence shedding light on the biological processes that enable dormant tumor cells to endure the multiple stresses encountered at the metastatic site.

## Introduction

Dormancy can be broadly defined as the process though which cells exit the cell cycle and survive in a quiescent state. Dormancy is considered an evolutionarily conserved mechanism of adaptation to stress which allows cells to survive in a hostile microenvironment. For instance, when the larvae of *Caenorhabditis elegans* are exposed to nutritional stress, a dormancy-like state, known as dauer diapause, is activated ^1, 2^. Memory T cells survive decades after entering a state of quiescence to protect an organism from recurrent infections. Proliferation of these cells resumes to produce effector T cells upon stimulatory signals ^3^. Likewise, mammalian adult stem cells are maintained in a physiological state of dormancy until appropriate signals, such as the loss of mature cells or the generation of a wound, trigger their proliferation ^4^. Dormancy not only is observed in physiological situations but also is known to play a key role in the course of cell transformation and tumor progression. Clinical tumor dormancy is thought to be the result of two possible mechanisms. One possible explanation is proliferative arrest, the inability of individual cancer cells to proliferate (cellular dormancy). Alternatively, dormancy may result from an equilibrium between proliferation and apoptosis that results in the equilibrium of a subclinical tumor mass (tumor mass dormancy) ^2^. Possible explanations proposed for mechanisms contributing to the balanced proliferative/apoptotic mechanisms include anti-tumoral activity of the immune system ^5, 6^ and the inability to induce an angiogenic network able to support tumor growth ^7^. Examination of autopsied specimens reveals the presence of *in situ* carcinoma in the prostate and breast from young, cancer-free individuals at frequencies of 34% and 39%, respectively, although the incidence of overt lesions at this age is much lower ^8, 9^. These and other findings have led to the concept of tumor latency preceding the development of the primary tumor ^10, 11^. This type of local tumor cell quiescence can be maintained for the lifetime of an individual, never leading to a symptomatic disease, or could be disrupted by yet-unknown mechanisms to initiate life-threatening primary tumor. Nevertheless, the relative contribution of tumor mass dormancy or cellular dormancy to tumor latency is yet unknown ^2^. However, tumor cell dormancy has been better characterized and studied in the context of metastasis.

Traditionally, metastasis has been considered a stepwise process through which cancer cells leave the primary tumor and invade surrounding tissues to finally intravasate blood vessels and disseminate to distant organs where they colonize. The dynamics of metastatic progression are highly unpredictable since this process can take from a few months to years and even decades to complete ^12, 13^. The long interval between the diagnosis and treatment of the primary tumor and subsequent appearance of metastatic disease suggests that disease may prevail in a dormant state ^2^.

Furthermore, recent data indicate that metastasis dissemination is a very early event occurring even before the primary tumor is detectable. Two recent articles provided complementary mechanisms of early dissemination in HER2-positive breast cancer ^14, 15^. Using a HER2-driven mouse model of breast cancer, Hosseini *et al*. ^14^ showed that progesterone signaling subsequent to HER2 activation promotes cancer cell migration in early breast cancer lesions, ultimately leading to the majority of metastatic lesions observed in this experimental model. In addition, Harper *et al*. ^15^ reported that the downregulation of p38 together with the expression of HER2 promoted the early dissemination of breast cancer cells (BCCs), which were shown to induce an epithelial-to-mesenchymal transition (EMT)-like invasive program dependent on Wnt. These early disseminated tumor cells (DTCs) were detected in the bone marrow and in the lungs of HER2-transgenic mice, where they either remained dormant for the duration of the experiment (bone marrow) or promoted the formation of metastatic lesions after a dormancy phase (lung). These and similar data derived from melanoma models ^16^, together with the identification of disseminated cells in the bone marrow of patients with early-stage breast cancer or even pre-invasive neoplasms such as ductal carcinoma *in situ* ^17^, suggest that there may be a much longer latency period between early metastatic dissemination and the detection of overt metastatic lesions than initially thought.

In this review, we will focus on metastatic tumor cell dormancy and the analysis of recent data aiming to characterize this process at a molecular level. Given that metastases are the main cause of cancer-related deaths ^18^, the elucidation of the molecular mechanism regulating the survival of DTCs in early stages of the metastatic cascade is critically important to develop effective therapeutic strategies against life-threatening metastatic disease.

## Microenvironmental regulation of disseminated tumor cell dormancy

As explained above, dormancy can be considered an adaptive response to microenvironmental stress. Several studies support the notion that DTCs enter a dormant state by being unable to establish appropriate interactions with the extracellular matrix (ECM) that support survival after infiltrating the parenchyma of metastatic target organs. Early evidence supporting this includes studies performed with the head and neck squamous carcinoma cells, HEp3, by the Ossowski laboratory ^19^. This study reported that urokinase plasminogen activator receptor (uPAR) downregulation induces tumor dormancy *in vivo* through the inhibition of the physical interaction between uPA/uPAR proteins with the α5β1 integrin, resulting in lower adhesion of the cells to fibronectin and lower mitogen-activated protein kinase/extracellular signal-regulated kinase (MAPK/ERK) pathway activation ^19^. In addition, this interaction activates the formation of insoluble fibronectin fibrils, which in turn inhibit p38 activity. Indeed, in the same article, the team showed that the proliferative status of HEp3 is dependent on a balance between the activities of ERK1/2 and p38 MAPKs. Elevated phosphorylation and activity of p38 result in cells entering a state of proliferative quiescence, whereas a switch toward ERK1/2 activation elicits cell proliferation ^20^. Remarkably, downregulation of uPAR in HEp3 cells inhibited focal adhesion kinase (FAK) phosphorylation and downstream Src activation promoting cellular dormancy *in vivo* ^21^. Shortly after, Liu *et al*. ^22^ reported the involvement of epithelial growth factor receptor (EGFR) in the activation of the uPAR/α5β1 integrin/fibronectin pathway in proliferating HEp3 cells. Additionally, the involvement of FAK and Src kinases in tumor dormancy and metastasis has been reported in other cancer models. Massagué *et al*. ^23^ reported that, when introduced into the bone marrow, MDA-MB-231 BCCs upregulate the expression of Src kinases as a pro-survival strategy. Activation of Src kinases positively regulates the phosphoinositide 3-kinase (PI3K)/AKT pathway in response to CXCL2/CXCR4 signaling and confers resistance to tumor necrosis factor–related apoptosis-inducing ligand (TRAIL), leading to cell survival and resistance to apoptosis. They also reported that Src knockdown had limited or no effect on lung tumor burden when using either MDA-MB-231 bone or lung metastatic derivative cell lines. Later, Nobutani *et al*. ^24^ corroborated these data by reporting that CXCR4 downregulation is involved in the maintenance of dormant MDA-MB-231 cells in the lung. These data highlight the specific role of Src in bone metastasis. Strikingly, Price *et al*. ^25^ used real-time *in vivo* microscopy of bone marrow in a breast cancer xenograft model to show that the CXCL2/CXCR4 interaction tethers BCCs to the bone marrow microenvironment and that CXCR4 inhibition releases micrometastasis into the circulation, therefore preventing metastatic progression. In addition, another study has shown the involvement of osteoclasts in the dormant-to-proliferative switch of dormant BCCs in the bone, where osteoclast progenitors are recruited to vascular cell adhesion molecule 1 (VCAM-1)-positive DTCs by interacting through integrin α4β1. This interaction promotes local osteoclast activity which effectively activates bone metastasis progression, although the specific mechanism through which dormant BCCs transition to a proliferative state after osteoclast recruitment and activation of osteoclasts was not explored ^26^.

Conversely, the activation of ITGB1 receptor leads to the formation of actin stress fibers and the proliferative outbreak of otherwise dormant BCCs. Barkan *et al*. found that the engagement of ITGB1 and downstream signaling through the activation of FAK, Src, ERK1/2, and myosin light-chain kinase (MLCK) was responsible for the dormant-to-proliferative switch of D2.0R mammary tumor cells both *in vitro* and *in vivo* ^27, 28^. In line with these initial data, el Touny *et al*. demonstrated that Src inhibition maintains quiescence, which in combination with ERK1/2 inhibition, elicits apoptotic cell death of dormant BCCs ^29^. Despite the limited knowledge about the molecular mechanisms modulating metastatic dormancy, these data highlight the importance of tumor cell interactions with the microenvironment, especially with components of the ECM, in the initial stages of cancer dissemination and metastatic tumor colonization.

In a later analysis, Aguirre-Ghiso *et al*. observed that p38 positively regulated the expression of p53, basic helix-loop-helix protein 3 (BHLHB3) (also known as DEC2 or SHARP1), and nuclear receptor subfamily 2 group F member 1 (NR2F1), which were essential for the maintenance of the dormant phenotype of HEp3 cells *in vivo* ^30^. This program is activated in dormant DTCs by transforming growth factor-beta 2 (TGF-β2) in the bone marrow, where it defines a metastasis-restrictive microenvironment ^31^. Specifically, NR2F1 has been reported to cooperate with TGF-β2 in the bone marrow, promoting DTC survival itself and quiescence via SRY-box 9 (SOX9), retinoic acid receptor beta (RARb), and NANOG ^32^. As mentioned earlier, the downregulation of p38 has been linked to the early dissemination of HER2-positive breast cancer ^15^. Notably, HER2-positive early DTCs were also shown to enter a p38-independent dormant state since systemic inhibition of p38 did not stimulate their proliferative outgrowth, as shown in other cancer models ^31, 33, 34^. These data suggest that dormancy in early DTCs might be governed by molecular mechanisms different from those regulating dormancy in more progressed tumors. In another study, the inhibition of the lysophosphatidic acid receptor 1, through increased p38 and decreased ERK1/2 activities, led to metastatic dormancy of 4T1 and MDA-MB-231T cells *in vivo* ^35^. The Src kinases Yes and Fyn have been shown to upregulate the expression of Claudin-2, through which metastatic BCCs disseminated to the liver physically interact with hepatocytes by means of heterotypic structures reminiscent of tight-junctional complexes that enable the formation of metastatic lesions in this organ ^36, 37^. Finally, the perivascular niche has been shown to strongly impact DTC dormancy. Ghajar *et al*. ^38^ found that the endothelium induced quiescence in breast DTCs through the production of thrombospondin-1 (TSP-1) but that sprouting neovasculature induced DTC proliferation mediated by the secretion of TGF-β1 and periostin (POSTN) by endothelial tip cells.

Finally, inflammation and immunity play key roles in tumor development and metastasis ^39^. An increasing body of evidence suggests that inflammation promotes the dormant-to-proliferative switch ^40^. As an example, the analysis of clinical data from 734 breast cancer patients who underwent successful treatment of their primary tumors showed a positive correlation between high levels of circulating acute-phase proteins and distant recurrence ^41^. Interestingly, recent work by de Cock *et al*. ^42^ links inflammation to the awakening of dormant DTCs through the expression of Zeb1, a well-known regulator of the EMT. On the other hand, tumor-infiltrating immune cells are important elements of the tumor microenvironment and have been shown to promote tumor cell dormancy ^43^. These mechanisms have recently been reviewed elsewhere and readers are referred to these articles for more information ^44, 45^.

Overall, these studies indicate that microenvironmental cues affecting DTC dormancy regulation may be specific to the metastatic target organ and the specific localization of the DTCs. Whether there are common core pathways affecting DTC growth or survival remains to be determined.

## Unfolded protein response and autophagy

Exploring the fundamental involvement of stress-induced pathways in metastatic dormancy has led to the notion that endoplasmic unfolded protein response (UPR) and autophagy might play an important role. Dormant HEp3 cells upregulate activating transcription factor 6 alpha (ATF6α), inositol-requiring enzyme 1 alpha (IRE1α), and protein kinase R-like endoplasmic reticulum kinase (PERK), which are the three major transducers of the UPR ^46, 47^. Among them, PERK has been shown to induce a strong G _0_-G _1_ arrest and promote cell survival *in vitro*. In addition, PERK overexpression was shown to inhibit tumor growth of colon carcinoma cells *in vivo*. The authors reported that these effects are mediated by the transcriptional repression of cell cycle regulators such as cyclin D1, cyclin D3, and cyclin A ^46^.

Further investigation of the role of UPR in dormant cell survival revealed that p38 upregulates the expression of endoplasmic reticulum (ER) chaperone BiP (Grp78) and PERK, promoting drug toxicity resistance and survival by preventing the activation of the pro-apoptotic protein Bax in dormant HEp3 cells ^34^. Overexpression of UPR proteins such as Grp78, Grp94, and protein disulfide-isomerase (PDI) has been observed in DTC cell lines established from DTCs found in the bone marrow of breast cancer patients with no clinical sign of metastasis ^48^. The active spliced isoform of X-box binding protein 1 (XBP-1), a downstream effector of IRE1α known to regulate the tumorigenicity and progression of triple-negative breast cancer, is enriched in tumor-initiating cells and cooperates in the transactivation of known hypoxia-inducible factor 1 (HIF1) targets ^49^. Silencing XBP-1 or ATF6α reduced the number of surviving HEp3 cells in a dormant state ^47^. Surprisingly, ATF6α was shown to induce the survival of HEp3 dormant cells through the activation of Akt-independent mammalian target of rapamycin (mTOR) signaling. This effect was mediated by the upregulation of the small GTPase Rheb, which directly activates mTOR-induced cell survival ^47^.

The involvement of autophagy in metastatic dormancy as an important stress-response mechanism has been proposed in the literature, although few experimental studies have directly addressed it. Early experimental evidence showing the involvement of autophagy in tumor dormancy was reported by Gupta *et al*. ^50^ in 2010. They observed that gastrointestinal stromal tumors (GISTs) treated with KIT/platelet-derived growth factor receptor A (PDGFRA) inhibitors, such as imatinib mesylate, are reduced to quiescent tumor cell subpopulations that are highly dependent on autophagy. The therapeutic combination of autophagy inhibitors and imatinib reduced the number of dormant GIST cells *in vitro* and *in vivo*, offering a promising therapeutic option for patients with GIST.

Later studies revealed that aplasia ras homolog member 1 (ARHI1) is a tumor suppressor and induces dormancy in xenografted ovarian tumors. The dormant state is fully reversible upon re-expression of ARHI. ARHI-induced growth arrest is concomitant with the upregulation of autophagosome formation and fully reversible upon ARHI re-expression. However, treatment of ovarian xenografts with the autophagy inhibitor chloroquine dramatically impaired the survival of growth-arrested ovarian cancer cells, suggesting that autophagy is a critical process which sustains tumor dormancy ^51^. In contrast to the data derived from dormant HEp3 cells, dormant SKOv3 ovarian cancer cells block PI3K signaling and inhibit mTOR to induce autophagy upon ARHI1 re-expression ^51^. Indeed, it is well known that deregulation of the PI3K/Akt/mTOR signaling pathway and activation of AMP-activated protein kinase (AMPK) activate autophagy. Autophagy can also be activated by UPR as aggregated misfolded proteins accumulate, exemplifying the intricate crosstalk between autophagy and UPR ^52^.

Although PI3K/Akt/mTOR pathway is constitutively activated in several types of tumors, AKT is often downregulated in dormant DTCs ^2, 53^. In fact, a highly active PI3K/AKT pathway has been shown to be essential for the metastatic outgrowth of MDA-MB-231 cells in the bone but to counteract the dormancy-promoting activity of p38 in MCF10A-HER2 BCCs ^15^. Interestingly, Dey-Guha *et al*. reported that MCF7 cells divide asymmetrically to generate a highly proliferating daughter cell and a slowly proliferating daughter cell, the latter resulting from the inhibition of AKT ^54^. The treatment of these cells with AKT inhibitors altered their asymmetric division toward the generation of the slow proliferating cell population, which is resistant to chemotherapy *in vitro* ^54^. The decrease in β1-integrin/FAK activity results in the asymmetric division of dividing MCF7 cells leading to the generation of a growth-arrested cell population. The authors reported that β1-integrin/FAK activation induced the mTORC2 complex but that AKT suppression via tetratricopeptide repeat domain 3 (TTC3) mediated proteasome degradation ^55^. mTORC2 activity, which is normally required for AKT activation, seems not to be involved in the proteasomal degradation of AKT in asymmetric division and provides another example of how mTORC signaling can be activated in dormant cells independent of AKT. The significance and function of the AKT-independent activation of the mTOR complexes in the context of tumor dormancy remain to be elucidated, but it exemplifies the complex molecular interactions often observed in the study of tumor dormancy.

An important question regarding UPR and autophagy in cancer cell dormancy is the importance of the activation of these processes in dormant DTCs and the consequences of their inhibition, which might have profound translational implications. A recent study by García-Prat *et al*. ^56^ shows that autophagy is essential to maintain a quiescent state in skeletal muscle stem cells. Autophagy impairment leads to senescence entry and loss of the regenerative capacity of the stem cells. Whether autophagy inhibition would eradicate dormant DTCs, force them into a non-reversible quiescent state, or just delay the dormant-to-proliferative switch and metastatic relapse remains to be determined.

## Hypoxia

Hypoxia has been extensively studied in the context of metastasis. It has been invariably linked to poor prognosis and tumor aggressiveness, but the underlying mechanisms have not been fully elucidated ^57^. Very recently, Fluegen *et al*. ^58^ showed a connection between exposure to hypoxia in the primary tumor and metastatic dormancy. The authors found that HEp3 cells and breast cancer MDA-MB-231 and ZR-75-1 cells subjected to hypoxic microenvironments in the primary tumor showed increased expression of key dormancy genes such as NR2F1, DEC2, and p27. This increase in the expression of dormancy genes was accompanied by the overexpression of HIF1 and glucose transporter 1 (GLUT1), known to play a central role in the cellular response to hypoxic conditions. Furthermore, the same expression profile was found in solitary DTCs in patient-derived xenograft (PDX) and transgenic MMTV polyoma middle T (MMTV-PyMT) mice. In addition, the data revealed that NR2F1 and HIF1 were essential for the expression of p27, which has been shown to promote cell cycle arrest in dormant DTCs ^27, 29, 30^. The authors argue that the data from this study identify the cell subpopulation responsible for cancer recurrence and poor prognosis associated with hypoxia. Another research group reported that subjecting MDA-MB-231 cells to several cycles of hypoxia and re-oxygenation generates a cell subline able to survive hypoxic conditions in a dormant state that is reversible upon normoxia exposure ^59^. In addition, the interleukin-6 (IL-6) cytokine leukemia inhibitory factor receptor (LIFR) and its downstream effector signal transducer and activator 3 (STAT3) activate suppressor of cytokine signaling 3 (SOCS3). The LIFR:STAT3:SOCS3 signaling pathway, which has been shown to be inhibited under hypoxia, downregulates the expression of quiescence and cancer stem cell–associated genes to promote the proliferation of otherwise dormant breast DTCs in the bone marrow. Therefore, this work indirectly links hypoxia with the outgrowth of dormant breast DTCs in the bone marrow, although the specific molecular mechanism activated in DTCs to trigger the dormant-to-proliferative switch remains to be explored ^60^. In lung cancer cells, hypoxia-induced expression of mitogen-induced gene 6 (MIG6) promotes cell quiescence through the inhibition of EGFR and downregulation of the PI3K/AKT and ERK1/2-mediated pathways ^61^. Finally, PI3K/AKT pathway inhibition through the blockade of its downstream effector mTOR is responsible for the reversible dormant state that human papillomavirus (HPV)-positive cervical and head and neck cancer cells enter upon hypoxia exposure ^62^.

Given these recent results and the conclusions drawn in the previous section, it would be of great interest to establish whether there is a functional relation between hypoxia and autophagy in cancer cell dormancy. Hypoxia-induced autophagy via BNIP3 and BNIP3L has been described as a stress-survival mechanism in mouse embryonic fibroblasts and various cancer cell lines to promote cancer progression ^63^. Exploring the possible crosstalk between these two mechanisms may provide a better understanding of DTC biology and open new research avenues for the development of effective therapies against cancer recurrence.

## Concluding remarks

Despite the improvement of our knowledge regarding the molecular mechanisms governing tumor dormancy and metastasis, this phase of tumor progression remains incompletely understood. This is primarily a consequence of the remarkable scarcity of models to study metastatic dormancy as well as the difficulties to detect minimal residual disease in patients with cancer. An open question in the field is whether it would be more advantageous to maintain DTCs in a permanent dormant state or to promote the dormant-to-proliferative switch in quiescent DTCs in order to kill them with conventional chemotherapy targeting the DTCs induced to proliferate. Obviously, the latter strategy involves the risk of inducing metastatic progression of the “awakened” DTCs that survive the treatment in a patient who might have otherwise remained disease-free.

Maintaining DTCs in a quiescent state indefinitely would transform cancer into a chronic manageable disease. However, the risk remains that the dormant cells might be triggered to proliferate in response to some stimulus. Thus, elimination of the dormant cell population would provide the more definitive solution. Recent advances in our understanding of how survival mechanisms enable quiescent tumor cells to survive the stresses inherent in tumor dissemination and distant colonization provide the opportunity to design new therapeutic strategies that target key components of these molecular pathways. Clinical trials based on this knowledge and their potential impact on the long-term survival of patients with cancer warrant further efforts.
